# Unraveling the genetic architecture of major depressive disorder: merits and pitfalls of the approaches used in genome-wide association studies

**DOI:** 10.1017/S0033291719002502

**Published:** 2019-09-27

**Authors:** I. Schwabe, Y. Milaneschi, Z. Gerring, P. F. Sullivan, E. Schulte, N. P. Suppli, J. G. Thorp, E. M. Derks, C. M. Middeldorp

**Affiliations:** 1 Department of Methodology and Statistics, Tilburg University, Tilburg, The Netherlands; 2 Translational Neurogenomics Laboratory, QIMR Berghofer Medical Research Institute, Brisbane, Australia; 3 Department of Psychiatry, Amsterdam Neuroscience and Amsterdam Public Health Research Institute, Amsterdam University Medical Center, Amsterdam, The Netherlands; 4 Department of Medical Epidemiology and Biostatistics, Karolinska Institutet, Stockholm, Sweden; 5 Department of Genetics, University of North Carolina, Chapel Hill, NC, USA; 6 Department of Psychiatry, University of North Carolina, Chapel Hill, NC, USA; 7 Medical Centre of the University of Munich, Munich, Germany; 8 Mental Health Centre Copenhagen, Copenhagen University Hospital, Copenhagen, Denmark; 9 Child Health Research Centre, University of Queensland, Brisbane, Australia; 10 Child and Youth Mental Health Service, Children's Health Queensland Hospital and Health Service, Brisbane, Australia; 11 Department of Biological Psychology, VU University Amsterdam, Amsterdam, The Netherlands

**Keywords:** Depression, GWAS, MDD, phenotypic heterogeneity, power, PRS, psychometrics

## Abstract

To identify genetic risk loci for major depressive disorder (MDD), two broad study design approaches have been applied: (1) to maximize sample size by combining data from different phenotype assessment modalities (e.g. clinical interview, self-report questionnaires) and (2) to reduce phenotypic heterogeneity through selecting more homogenous MDD subtypes. The value of these strategies has been debated. In this review, we summarize the most recent findings of large genomic studies that applied these approaches, and we highlight the merits and pitfalls of both approaches with particular attention to methodological and psychometric issues. We also discuss the results of analyses that investigated the heterogeneity of MDD. We conclude that both study designs are essential for further research. So far, increasing sample size has led to the identification of a relatively high number of genomic loci linked to depression. However, part of the identified variants may be related to a phenotype common to internalizing disorders and related traits. As such, samples containing detailed clinical information are needed to dissect depression heterogeneity and enable the potential identification of variants specific to a more restricted MDD phenotype. A balanced portfolio reconciling both study design approaches is the optimal approach to progress further in unraveling the genetic architecture of depression.

## Introduction

The advent of the genomic era represents a turning point in unraveling the biological underpinnings of depression. The term depression, if not otherwise specified, is used throughout this review in its broadest meaning, from relevant symptoms assessed via self-report methods to clinical diagnosis ascertained by psychiatric interview. Specific definitions adopted in different studies will be described when discussing the related results. The heritability estimate for major depressive disorder (MDD), defined as psychiatric diagnosis established according to the criteria based on the *Diagnostic and Statistical Manual of Mental Disorders* (DSM, American Psychiatric Association, 2000, 2013) or the *International Statistical Classification of Diseases and Related Health Problems* (ICD, World Health Organization, 2018), is around 40% (Sullivan *et al*., 2000). Initial failures to reliably detect associations of single genetic variants with MDD were attributed to underpowered studies with small sample sizes and to the clinical heterogeneity of the psychiatric trait, further compromising the power of association studies. Levinson *et al*. (2014) summarized the challenges facing the initial genome-wide association studies (GWAS) of MDD and proposed two, non-mutually exclusive, strategies to overcome them.

The first suggestion was to maximize the number of cases and controls, a strategy that has been successfully applied to schizophrenia (Schizophrenia Working Group of the Psychiatric Genomics Consortium, 2014) and many other complex diseases (Visscher *et al*., 2017). Due to the higher prevalence and lower heritability of MDD, it was estimated that three to five times as many cases would be required to detect the same number of genome-wide significant single-nucleotide polymorphisms (SNPs) as compared to schizophrenia (Wray *et al*., 2012). The second proposed strategy was to enhance GWAS statistical power by reducing heterogeneity through selecting clinically more homogenous depression phenotypes.

In recent years, both strategies have been applied. To increase power by increasing sample size, subjects with self-reported diagnoses for depression and/or continuous measures of the whole phenotypic range of depression were included in the analyses (e.g. Wray *et al*., 2018). An advantage of this approach is that this leads to an increased sample size without having to face the logistical and financial challenges of collecting large clinical MDD samples. However, the validity of this procedure has been criticized (Abbasi, 2017). For example, it has been argued that a self-reported clinical diagnosis cannot be transferred one to one to a psychiatric assessment, resulting in the inclusion of misclassified and/or clinically non-relevant cases.

To increase power by decreasing heterogeneity, studies have been specifically designed to recruit depression cases with more severe profiles (e.g. with recurrent MDD or with diagnosis made in hospital settings) (CONVERGE consortium, 2015; Pedersen *et al*., 2018). Other researchers have tried to decrease heterogeneity by stratifying MDD patients according to relevant clinical features, such as age of onset, symptom profiles, or postpartum depression (Viktorin *et al*., 2016; Milaneschi *et al*., 2017; Power *et al*., 2017). A relatively small number of replicated loci have been identified in the studies that applied these procedures.

Greater awareness of the merits and pitfalls of both strategies is instrumental for their effective application in the next generation of studies aimed at advancing our understanding of the genetics of depression. This review summarizes the evidence emerging from the application of the two strategies in the context of large genomic studies. After reporting the most recent findings, we highlight the main strengths supporting their rationale and major points of criticism, with a particular attention to methodological and psychometric issues. The review concludes with a discussion of future opportunities and challenges.

## GWAS in depression

Table 1 gives an overview of all GWAS on depression that has been published so far. The table is restricted to GWAS with a sample size of at least 10 000, but a complete list can be found in the online Supplementary material. For every study, details of the study population and the used assessment instrument(s) are provided. Different definitions of depression were used across different studies, based on clinical diagnosis, self-reported clinical diagnosis, or self-reported symptom/questionnaire data. For example, the first study that successfully identified genetic variants for depression selected a population with a relatively homogeneous phenotype. The CONVERGE consortium restricted the phenotype to recurrent severe MDD (patients from clinical settings with at least two episodes) in Han Chinese women (5303 cases and 4337 controls). Two independent and replicable genetic risk loci were significantly associated with this phenotype (CONVERGE consortium, 2015). Secondary analyses were restricted to cases who met the DSM-IV criteria for melancholia (4509 cases and 5377 controls), a more severe subtype of MDD (Kendler, 1997). Although this sample was smaller, the association with the two significant variants was found to be stronger. So far, these associations have not been identified in European samples (Major depression Working Group of the Psychiatric GWAS consortium *et al*., 2013; Wray *et al*., 2018), possibly because these variants occur at low frequency in individuals of European ancestry.

**Table 1.** Overview of the number of significant loci and *H*^2^_SNP_ in genome-wide association studies on depression (sample size >10 000 subjects)

Study	Population	Depression phenotype/s^a^ (main cohorts included)	Ascertainment			*N* Total	*N* cases	*N* controls	GWS loci	*H*^2^_SNP_ (s.e.)
			Clinical diagnosis/diagnostic interview	Self-reported diagnosis/treatment	Self-reported questionnaires/symptoms					
Kohli *et al*. (2011)	European	MDD (MARS plus 6 additional replication cohorts)	x			15 089	4088	11 001	0	*
Wray *et al*. (2012)	European	MDD (MDD2000 + plus 2 additional cohorts)	x			12 664	5763	6901	0	*
PGC-MDD (2013)	European	MDD (PGC-MDD)	x			18 759	9240	9519	0	*
Hek *et al*. (2013)	European	Depressive symptoms (CHARGE)			x	34 549	−	−	0	*
CONVERGE consortium (2015)	Han Chinese women	Recurrent MDD (CONVERGE)	x			10 640	5303	5337	2	0.21 (0.030)
Okbay *et al*. (2016)	European	Depressive symptoms (UK Biobank + PGC-MDD)	x		x	180 866^b^	16 471	58 835	2	0.04 (0.004)
Hyde *et al*. (2016)	European	Major depression (23andMe + PGC-MDD)	x	x		478 240	130 620	347 620	15	0.06 (*)
Direk *et al*. (2016)	European	Broad depression meta-analysis of PGC-MDD (2013) and Hek *et al*. (2013)	x		x	70 017^b^	9240	9519	1	0.30 (0.040)
		MDD	x			18 759	9240	9519		0.21 (0.020)
		Depressive symptoms			x	51 258	–	–		0.04 (0.010)
Power *et al*. (2017)	European	Age at onset stratified MDD:	x			18 439	8920	9519		
		Late-onset (adult-onset) MDD (PGC-MDD)	x			~ 13 519 (octiles 5-8)	~4000	9519	1	0.23 (0.046)
Milaneschi *et al*. (2017)	European	MDD (all cases)	x			26 628	11 837	14 791	na	0.14 (0.08)
		MDD with increased appetite/weight	x			16 662	1871	14 791	0	0.11 (0.03)
		MDD with decreased appetite/weight	x			20 138	5347	14 791	0	0.11 (0.02)
		MDD with no change in appetite/weight (PGC29)	x			18 212	3421	14 791	na	0.08 (0.02)
Hall *et al*. (2018)	European	Major depression (all cases)	x	x	x	43 062	10 851	32 211	0	0.12 (0.02)
		Recurrent major depression	x	x	x	39 556	7345	32 211	0	0.12 (0.02)
		Male major depression	x	x	x	19 886	3852	16 034	1	0.13 (0.03)
		Female major depression (UK Biobank + Generation Scotland)	x	x	x	23 169	6997	16 172	0	0.05 (0.03)
Peterson *et al*. (2018)	Han Chinese women	Recurrent MDD (all cases)	x			9599	4785	4814	0	0.31 (0.037)
		Recurrent MDD with exposure to adversity	x			2628	1646	982	0	0.34 (0.159)
		Recurrent MDD with no exposure to adversity (CONVERGE)	x			6971	3139	3832	3	0.38 (0.048)
Howard *et al*. (2018)	European	Help-seeking for mental health difficulties (Broad depression)		x		322 580	113 769	208 811	14	0.10 (0.004)
		Probable major depression		x	x	174 519	30 603	143 916	2	0.05 (0.006)
		MDD (ICD-coded) (UK Biobank)	x			217 584	8276	209 308	1	0.10 (0.012)
Wray *et al*. (2018)	European	Major depression (PGC29 + 23andMe + UK Biobank + Generation Scotland + 3 additional cohorts)	x	x	x	461 134	135 458	344 901	44	0.09 (0.004)
Li *et al*. (2018)	European (*n* = 326 113) and Han Chinese women (*n* = 10 640)	Major depression meta-analysis of Hyde *et al*. (2016), Ripke *et al*. (2013), and Cai *et al*. (2015)	x	x		336 753	90 150	246 603	10	*
Dunn *et al*. (2018)	Hispanic/Latino	Depressive symptoms			x	12 310	–	–	0	0.04 (0.031)
		Depressive symptoms adjusted for anti-depressant use			x	12 310	–	–	0	0.03 (0.031)
		Depressive symptoms excluding anti-depressant users (HCHS/SOL)			x	11 486	–	–	0	0.04 (0.033)
Howard *et al*. (2019)	European	Major depression meta-analysis of Hyde *et al*. (2016), Howard *et al*. (2018), and Wray *et al*. (2018)	x	x	x	807 553	246 363	561 190	101	0.09 (0.003)
Cai *et al*. (2018)	European	Help-seeking from psychiatrist		x		333 412	36 286	297 126	5	0.13 (0.018)
		Help-seeking from GP		x		332 622	113 260	219 362	24	0.14 (0.008)
		Probable major depression		x	x	79 575	21 117	58 398	0	0.18 (0.015)
		Self-reported major depression		x		253 919	19 805	234 114	0	0.11 (0.009)
		DSM-based major depression			x	67 171	16 301	50 870	1	0.26 (0.022)
		Recurrent DSM-based major depression (UK Biobank)			x	59 385	10 302	49 083	0	0.32 (0.026)

MARS, Munich Antidepressant Response Signature project; PGC-MDD, Major Depressive Disorder Working Group of the Psychiatric Genomics Consortium; CHARGE, Cohorts for Heart and Aging Research in Genomic Epidemiology consortium; CONVERGE, China, Oxford and Virginia Commonwealth University Research on Genetic Epidemiology consortium; HCHS/SOL, Hispanic Community Health Study/Study of Latinos.

*Not reported.

a ‘MDD’: ascertainment by clinical diagnosis or diagnostic interview fulfilling the criteria for major depressive disorder; ‘major depression’: ascertainment by self-reported diagnosis or treatment for major depressive disorder; ‘depressive symptoms’ phenotypes that utilize self-reported symptoms of major depression.

b *N* total includes the number of individuals in cohorts with continuous measures as well as the total number of cases and controls.

Another group of studies tried to capitalize on maximizing sample size to identify significant genetic variants. For example, in order to assemble very large samples to increase statistical power, a number of collaborative studies combined data that relies on instruments based on self-report, potentially resulting in large differences in phenotypic depth among the different combined samples. At the time of writing, one of the largest published GWAS meta-analyses consisted of 135 458 cases and 344 901 controls (Wray *et al*., 2018). The data structure included an ‘anchor PGC29 cohort’ from the PGC combining 29 samples using mostly standard methods for assessing lifetime MDD (i.e. personal interviews by trained interviewers using structured diagnostic methods). These data were combined with those of six ‘expanded’ cohorts that used different methods to identify clinically-significant depression: deCODE, GERA, and iPSYCH used electronical medical records, Generation Scotland structured diagnostic interview for MDD, UK Biobank both self-report of symptoms or help-seeking and electronic records, and 23andMe self-reported clinical diagnosis or treatment by a medical professional. Subjects meeting MDD formal clinical criteria and those self-identified with minimal phenotyping were classified as cases of a broader phenotype labeled ‘major depression’ (MD). The GWAS identified 44 genome-wide significant independent loci (Wray *et al*., 2018). More recently, this GWAS was combined with the latest data released from UK Biobank, which included a phenotype labeled ‘broad depression’ which was based on two self-report questions on help-seeking for mental health difficulties, totaling 246 363 cases and 561 190 controls (Howard *et al*., 2019). This resulted in the identification of 101 independently associated loci at a genome-wide significant level.

## Studies on heterogeneity

With the exception of CONVERGE, no large GWAS has investigated genetic risk factors of a more homogenous MDD definition, but various studies have aimed at dissecting depression heterogeneity by analyzing existing large-scale genomics datasets. One of the approaches used so far is to stratify cases along relevant clinical features, which are then compared in terms of their genetic profile to establish whether they form more homogenous subgroups.

The selection of clinical features relevant for stratification, so far, has been based on the results of family and twin studies. Early-onset MDD, defined as MDD with the first episode taking place before the age of 30 years, has been observed to be associated with a higher risk of MDD in relatives, while late onset has been related to a higher risk of vascular diseases in relatives (Kendler *et al*., 2009). Furthermore, two major twin studies have found a higher heritability for MDD in women than in men (approximately 40% *v.* 30% and 42% *v.* 29%, respectively) and clear evidence for sex-specific genetic effects with an estimated genetic correlation in liability to MD in men and women at approximately 0.55 and 0.63, respectively (Kendler *et al*., 2001, 2006). Moreover, unaffected co-twins of patients endorsing atypical ‘reversed vegetative symptoms’ (e.g. hyperphagia, weight gain, and hypersomnia) had a higher body mass index (BMI) (Kendler *et al*., 2009). Lastly, the differential impact of environmental risk factors has been considered to be another major source of heterogeneity, as the variance in liability to MDD has a comparatively large environmental component (Sullivan *et al*., 2000).

Analyses based on GWAS results showed moderate to high genetic correlations between subgroups of cases stratified according to the clinical features listed above. A genetic correlation, commonly denoted as *r*_g_, represents a correlation between the true effect sizes of SNPs affecting the different subgroups (or two different traits). A high correlation implies that, on average, SNPs have directionally similar effects on the two subgroups (see, e.g. Maier *et al*., 2018 for more details). In data from the anchor cohort of the PGC, *r*_g_ between early-onset and late-onset MDD was approximately 0.99 (Power *et al*., 2017), and around 0.82 between MDD patients with atypical symptoms of increased appetite and/or weight and those with more typical symptoms of decreased appetite and/or weight (Milaneschi *et al*., 2017). In data from CONVERGE, *r*_g_ between MDD cases exposed to early stressful life events and childhood sexual abuse and unexposed cases was around 0.62 (Peterson *et al*., 2018).

However, the results of polygenic risk score analyses also indicated genetic heterogeneity. In PGC data, cases with earlier-onset MDD had a higher polygenic risk for schizophrenia and bipolar disorder (Power *et al*., 2017). After stratification by age of onset quantiles, one replicated genome-wide significant locus for the oldest quartile (adult-onset, >27 years) was identified. Furthermore, also in PGC data, MDD patients with atypical increased or typical decreased appetite and/or weight were divergent in the extent of overlap with genetic variants for immune-metabolic features: only MDD with atypical symptoms showed a specific overlap (*r*_g_ = 0.53) with BMI (Milaneschi *et al*., 2017). Compared to the control group, only depressed patients with atypical symptoms carried significantly higher polygenic risk burden for increased BMI, and circulating high levels of CRP, leptin, and BMI-adjusted leptin. In stratified GWAS analyses, the direct comparison between the two subgroups of cases yielded one genome-wide significant association. Lastly, to estimate the degree of heterogeneity due to exposure to adversity, the genomic relationship matrix (GRM) of the CONVERGE data was extended to include an interaction with a measure of adversity exposure (e.g. early stressful life events and childhood sexual abuse) in the SNP-based heritability 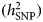 estimation. The GRM estimates the genetic relationship between individuals based on SNP information and 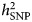 denotes the variance explained by all SNPs used in a GWAS in conventionally unrelated individuals (Yang *et al*., 2017). Results suggested that 13.2% of MDD liability could be attributed to genome-wide interaction with adversity exposure. Furthermore, an adversity exposure-stratified GWAS comparing MDD cases against controls detected three associated loci only in participants with no history of adversities. Appropriate replication was however not feasible due to unavailability of samples with similar features (Peterson *et al*., 2018). Sex-stratified analyses in the UK Biobank and Generation Scotland: Scottish Family Health Study (GS:SFHS) yielded a similar 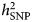 estimate (~0.2) across males and females and showed no detectable discrepancies in genetic overlap with health-correlated traits, but stratified GWAS analysis revealed one, non-replicated, genome-wide significant locus for MDD in male patients (Hall *et al*., 2018).

Another strategy to investigate heterogeneity is to apply an analytical technique called *Buhmbox*, which aims to verify whether the genetic correlation between two traits can be explained by the presence of a subgroup in the first trait (i.e. heterogeneity) that is genetically similar to the second trait (Han *et al*., 2016). This method was applied in preliminary analyses on >30 000 samples from UK Biobank and GS:SFHS showing that genetic correlations of MDD with high triglycerides, cholesterol, and blood pressure might be explained by heterogeneity among MDD cases. This provides further evidence for the presence of a subgroup of MDD cases in which metabolic alterations may represent a specific pathophysiological pathway (Howard *et al*., 2018).

## Merits and pitfalls of the two approaches

The strategy of maximizing sample size by expanding the phenotype of MDD as defined by the DSM or ICD to include self-reported diagnosis of depression and/or continuous measures of depression is based on the assumption that the underlying liability to depression is a normally distributed severity continuum in the population – with MDD representing the extreme tail of this distribution. The demarcation line that delineates MDD is thus somewhat arbitrary and not being an entirely separate entity that is in accordance with the ‘multiple-threshold model’ (Reich *et al*., 1972), asserting that different syndromes reflect only different levels of severity on a single dimension, not distinct etiologies. Under this conceptualization, combining different measures of depression, also when ‘sampled’ at different points in the underlying distribution (at the normal and at the clinical range) substantially increases the statistical power to detect common risk variants. Evidence for the multiple threshold model was found in a recent twin study showing that MDD and minor depression (characterized by at least two but fewer than five of the symptoms of MDD) lie on the same single dimension of liability with different levels of severity (Corfield *et al*., 2017). It is important to note that the study by Corfield *et al*. (2017) considered two similar clinically-ascertained syndromes mainly differentiated by the number of endorsed symptoms.

In contrast, it has been argued that depression diagnoses phenotypes obtained with clinical *v.* self-report assessment represent different entities rather than different thresholds on the same liability, and this is indicated by the difference in 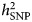 estimates for the different phenotypes. In particular, lower 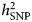 estimates obtained using self-report phenotyping have been considered as a result of misclassification of subjects with other conditions than depression (Cai *et al*., 2018). In the PGC GWAS (Wray *et al*., 2018), 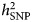 estimated on the liability scale for depression varied from 0.26 in the GenScot cohort (clinically ascertained) to 0.08 in 23andMe data (self-report), although the confidence intervals largely overlapped. In a preliminary analysis of UK Biobank data, Cai *et al*. (2018) reported an SNP-heritability of 26% for a DSM-based diagnosis of lifetime MDD derived from an online Mental Health questionnaire, and lower 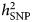 estimates (<15%) for alternative definitions based on minimal phenotyping. Previous results of the UK Biobank study (Howard *et al*., 2018) on the other hand did not reveal highly divergent 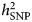 between different depression phenotypes, with estimates of ~10% for self-report broad depression and ICD-9 or 10 coded MDD based on hospital records. Note that the findings of lower heritability estimates of MDD based on self-report symptom/questionnaire data could possibly also be explained by an attenuation in heritability estimates due to random noise (e.g. higher unsystematic measurement error) resulting from a more noisy phenotype (van den Berg *et al*., 2007; van der Sluis *et al*., 2010; Schwabe *et al*., 2014, 2019) rather than from measuring a different phenotype.

Those in favor of the strategy of combining different depression phenotypes consider the presence of a strong genetic correlation between depression phenotypes measured with different instruments as empirical evidence of the validity of this approach. For example, in the PGC GWAS meta-analysis of major depression (Wray *et al*., 2018), the genetic correlations for the clinically-ascertained PGC29 cohort varied from 0.97 in the deCODE sample (including cases from electronical medical records) to 0.67 in the 23andMe sample (self-report assessment) and the weighted mean of all pairwise genetic correlations between cohorts was high (weighted mean *r*_g_ = 0.76, s.e. = 0.03). A formal test of heterogeneity between *r*_g_ estimates was found to be statistically non-significant, and the authors interpreted this result as an indication of a strong overlap in the common genetic architecture of the different phenotypes supporting the comparability of assessment via different measurement methods. Furthermore, Wray *et al*. (2018) found very high genetic correlations (close to +1) between the MD phenotype of their GWAS meta-analysis and two previous GWAS that focused on current depressive symptoms measured with self-report-based questionnaires. Similar high genetic correlations between clinically defined cases and current symptoms in the general population have been reported for other disorders such as ASD, ADHD, and OCD (Middeldorp *et al.*, 2016; Martin *et al*., 2018).

Nevertheless, others argue that the reported genetic correlations may arise mainly from the overlap across different depression phenotypes of a large portion of non-specific liability to poor mental health (Cai *et al*., 2018). The finding of high genetic correlations with different traits such as neuroticism and anxiety (Okbay *et al*., 2016; Cai *et al*., 2018; Wray *et al*., 2018) may be interpreted as emerging from shared non-specific vulnerability, while the multicomponent construct of MDD may be also influenced by *specific* genetic risk factors. As a consequence, the broad phenotype of MDD used in recent GWAS (Wray *et al*., 2018; Howard *et al*., 2019) may result in a substantive increase of power to detect shared genetic risk variants, but not be suitable to detect *specific* risk factors for MDD (McIntosh *et al*., 2019). Furthermore, the validity of interpreting the genetic correlation as a parameter to establish equivalency between phenotypes has been questioned. Some have highlighted the need to, instead, focus on the more conservative alternative of using the squared value (i.e. *r*_g_^2^) reflecting the percentage of SNP effects on one phenotype that can be *explained* by the SNP effects on other phenotypes (Cai *et al*., 2018).

**Fig. 1.**
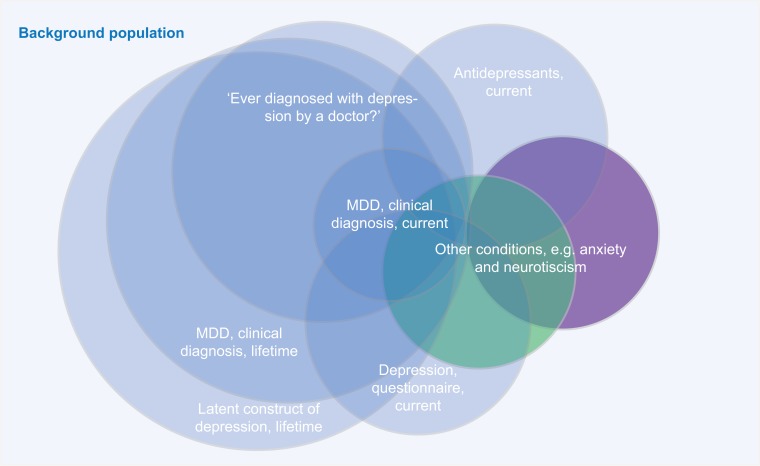
A major point of criticism of combining different depression diagnosis phenotypes is that different assessment methods might identify different parts of the ‘latent depression’ population.

Overall, the major point of concern raised about combining different depression diagnosis phenotypes (e.g. clinical ascertained diagnoses, self-reported diagnoses/treatment, or self-report questionnaires) might not capture the same quantitative or qualitative psychopathological entity (see Fig. 1 for an illustration of this issue).

Instead, MDD might underlie a distinct construct and misclassification can occur when participants state ‘yes’ to the question whether they have ever experienced a depression without ever having fulfilled the criteria for MDD. Their symptoms, for instance, may better fit with conditions with overlapping clinical features, such as dysthymia, anxiety disorders, somatic illnesses, substance use, and even normal bereavement. An in-depth clinical assessment, through detailed differential diagnoses probing, will provide different results than a self-reported clinical diagnosis (e.g. endorsing the question ‘Have you ever been diagnosed for depression’) or a diagnosis based on a questionnaire (e.g. a self-reported symptom score based on a cut-off). Similarly, the self-report of the indication for antidepressant use may represent a poor proxy for actual MDD. These concerns are strengthened by the results of some studies, showing for instance poor agreement between self-report *v.* psychiatry-lead interviews (Eaton *et al*., 2000), or that the majority of US adults that are or were treated with antidepressants did not actually screen positive for MDD (Olfson *et al*., 2016). Furthermore, differences between clinical interview and self-report measures may emerge from the different timeframe of the assessments: while lifetime is the timeframe for clinical diagnosis, measures based on self-report often focus on *current* symptoms.

One final issue closely related to the possibility of misclassification is that combining phenotypic data from multiple consortia generally comes with a number of psychometric issues at the phenotypic level. For example, aggregating questionnaire data from different consortia will most likely result in a violation of *measurement invariance*, meaning that the perception of the (severity) of the symptoms might depend on factors other than the severity of the illness-like properties of the questionnaire (e.g. choice of items or wording) or characteristics of the respondent that are not relevant for the disease (e.g. cultural or language background) (van den Berg *et al*., 2014).

**Fig. 2.**
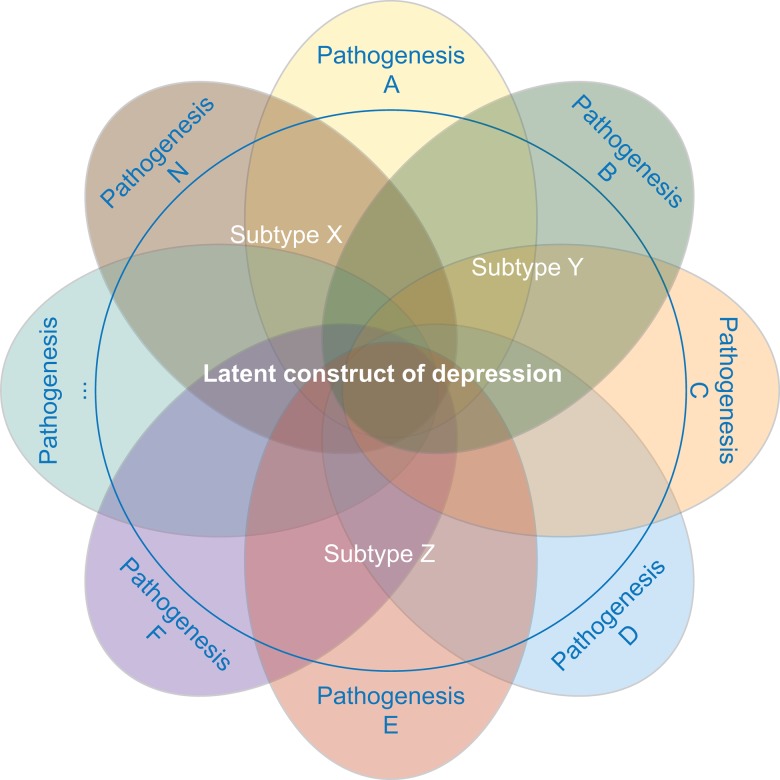
MDD is likely caused by multiple different etiopathological mechanisms. Studies investigating distinct subtypes of depression aim at reducing the underlying pathophysiological heterogeneity.

The strategy focused on selecting a more homogeneous depression phenotype is based on the assumption that the clinical heterogeneity in depression may emerge from an aggregation of different underlying liabilities expressed through partially distinct biological pathways (see Fig. 2 for an illustration).

Researchers who have applied this strategy argue that narrowing the phenotype and increasing phenotypic homogeneity may tag higher underlying genetic homogeneity and, hence, heritability, compensating for the related drop in sample size. This approach has been shown to be effective in the CONVERGE study that focused on recurrent severe depression in Han Chinese women and resulted in two significant hits. Furthermore, polygenic analyses provide evidence for the existence of heterogeneity in the depression phenotype. Nevertheless, applying this strategy can be challenging. First, the best criterion for stratification, allowing to accurately identify more homogenous subtypes, is a matter of debate. Second, few large genomic datasets include complete measures of the potential features of interest, such as specific symptom profiles. Overall, while different studies showed an increase in point-estimates for 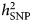 – in particular when moving from self-report-based instruments to clinically-ascertained depression and in some instances when stratifying MDD cases for certain features – the confidence intervals surrounding those estimates did not allow to formally confirm that the differences in 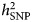 were of statistical significance. An under-investigated area in this respect is the use of repeated measures. Twin studies have shown that the stability in depression is largely explained by genetic factors (see, e.g. Nivard *et al*., 2015). Moreover, the factor reflecting the stability or agreement over measures has been reported to have a higher heritability than the individual measures (Foley *et al*., 1998; Lubke *et al*., 2016). Cheesman *et al*. (2018) estimated twin and 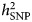 of a stable emotional problems phenotype that was constructed based on 12 measures from three ages and three raters using confirmatory factor analysis and item response theory modeling. They found that SNP heritability rose from 5% (not significant) on average for individual measures to 14% (s.e. = 0.049; *p* = 0.002) by focusing on stable trait variance.

## Discussion

Genetic discoveries in depression have lagged behind for a long time, due to several challenges unique to this phenotype (e.g. modest heritability, a high prevalence, the role of environmental influences, and phenotypic heterogeneity). To forward the field and increase power to find meaningful genetic associations, Levinson *et al*. (2014) proposed to apply two different non-mutually exclusive strategies to accelerate genetic discovery for depression: (1) to substantially increase the sample size of GWAS and (2) to reduce phenotypic heterogeneity by selecting clinically more homogenous subgroups of cases. The present review summarized the main findings, and the strengths and pitfalls of these two strategies.

In the light of the discussion to which extent the different depression phenotypes measure the same entity, it is important to acknowledge that we cannot directly observe MDD as we can other human characteristics (such as a person's height or someone's hair color). Consequently, in order to make MDD measurable, we need to specify an underlying (psychometric) model to operationalize it. As for every other latent (unobservable) psychiatric trait, the ‘correct’ model is unknown and we can adopt different frameworks. Those in favor of broadening the phenotype adopt a model that suggests that the difference in MDD among people is a matter of degree (e.g. MDD being represented by a dimension on which people can be ordered). On the other hand, those in favor of decreasing heterogeneity adopt a model where the difference in MDD is a matter of ‘type’ one belongs to or not (e.g. MDD being a different entity than other depressive disorders or subthreshold). This is an enduring issue in psychology (can psychological attributes be best represented as dimensions or categories?) that further complicates choosing between the two strategies. The findings that are discussed in this review provide evidence for both frameworks: strong and consistent genetic correlations across studies using different depression phenotypes provide empirical support for a common underlying internalizing trait and specific polygenic signatures for subgroups of depressed patients provide empirical support for the presence of distinct pathophysiological processes acting under the same diagnostic label. Overall, results from large-scale genetic studies draw a composite picture of the underlying liability of depression.

The aforementioned debate is closely related to what has been referred to as the ‘reification problem’ by Kendler (2014) stating that diagnostic criteria have been misinterpreted as the actual dimension they are designed to assess. The definition of MDD is not based on a fundamental biomarker or pathophysiology. Consequently, the diagnostic criteria currently used to evaluate the presence of MDD or, for that matter, any other psychiatric disorder, are based on descriptive signs, often selected from clinical tradition rather than from empirical evidence. In the absence of biological markers reflective of etiopathological mechanisms, major updates of psychiatry nosology have, so far, been revolving around the debate whether psychiatric disorders should be conceptualized as fewer broad categories or as more fine-grained categories. As Kendler (2014) highlighted, the absolute reliance on these criteria and the loss of awareness of their indexical function leads to reification and diagnostic literalism, confusing diagnostic criteria with the actual trait they are designed to assess.

Despite indications of detectable heterogeneity, so far the studies applying the strategy aimed at maximizing sample sizes identified a higher number of genome-wide replicated loci compared to those that focused on increasing homogeneity. The number of significantly associated genetic variants has been steadily increasing with increasing sample sizes, indicating that combining different depression phenotypes increases power to detect a large number of common genetic variants shared across similar but distinct phenotypes. In contrast, with the exception of CONVERGE, no other GWAS so far identified specific genetic variants reliably associated with MDD phenotypes defined more strictly. Given the high co-morbidity between MDD and other psychiatric disorders, such as anxiety disorders (Kessler *et al*., 2003) and personality traits such as neuroticism, and the prediction of MDD by subthreshold depression (Lee *et al*., 2018) these identified genetic variants may not be specific for MDD, but may be shared with the vulnerability for anxiety disorders, other depressive disorders, such as dysthymia and with subthreshold symptoms. We think that identifying these variants is evenly useful as identifying variants underlying specific more homogeneous MDD subtypes. This discovery base may be subsequently leveraged to disentangle divergent genetic effects for specific traits.

The availability of increasing numbers of samples with genotype–phenotype data in expanding cohorts and biobanks do provide the opportunity to apply different approaches without decreasing or even increasing power and also give justice to the heterogeneity. These approaches make the most efficient use of all data collected in the samples. For instances, the results of recent papers leveraging UkBiobank data suggest that specific genetic variants differentially influence individual items used to asses neuroticism (Nagel *et al*., 2018) and depressive symptoms (Thorp *et al*., in press). Population-based birth, child, and adolescent cohorts also provide excellent resources to make optimal use of the already available data by investigating genetic variants that influence stability over time (Middeldorp *et al*., 2019).

Recent initiatives introduced the application of efficient and scalable electronic instruments to assess psychiatric disorders, which may reconcile the need of both a larger number of persons screened and a more refined phenotyping of the trait of interest. For example, the comprehensive online mental-health questionnaire used in UK Biobank identifies operationally defined syndromes such as lifetime depression, mania, anxiety disorder, psychotic-like experiences and self-harm, post-traumatic stress disorder, and substance use disorders. Another example is the Lifetime Depression Assessment Self-report (LIDAS, Bot *et al*., 2017), which is largely based on the Composite International Diagnostic Interview (CIDI) short form for lifetime depression (CIDI-SF; Kessler *et al*., 1998; Hamilton *et al*., 2011) and assesses lifetime history of MDD according to DSM criteria. In feasibility studies, the LIDAS has shown adequate sensitivity (0.85) and specificity (0.80), and has a short median completion time (Bot *et al*., 2017). These tools hold the potential to provide reliable measures of depression at low cost in large (existing) cohorts and biobanks with genetic data. Furthermore, such instruments may provide information on specific clinical features along which cases can be stratified to identify more homogeneous subgroups, such as endorsement of single or specific combinations of symptoms. An important application of genetic stratification may also involve trials testing the efficacy of different treatments for depression: the identification of interactions between specific genomic risk profiles of various traits only with a certain class of treatments may provide interesting insights in the complex underlying pathophysiological mechanisms active in depression.

Finally, further developments in analytical methods and knowledge on depression heterogeneity may enhance the strengths of the two main strategies reviewed here. For instance, by leveraging the identified shared genetic liability across different phenotypes, the newly developed multi-trait analysis of GWAS (MTAG) has been shown to substantially increase statistical power to detect genetic association for each trait in a joint analysis of multiple traits using GWAS summary statistics (Turley *et al*., 2018). Pooling data on depression, neuroticism, and subjective well-being, the application of MTAG substantially increased the number of significantly associated loci for each trait, compared to single-trait analyses, with depression-associated loci going from 32 to 64 loci. Recently, multivariate methods were proposed that allow the identification of variants with effects on common cross-trait liability and variants that cause divergence, such as genomic structural equation modeling (GenomicSEM, Grotzinger *et al*., 2019) and genome-wide association meta-analysis (GWAMA, Baselmans *et al*., 2019).

To conclude, so far increasing the sample size by carefully and thoughtfully adding samples with non-traditional diagnostic approaches has enabled the identification of a large number of genetic variants for depression, but this must be done with great care. Part of these variants probably also influence other internalizing disorders and related traits. As sample sizes increase, further increases in the number of genetic loci robustly associated with depression will likely be achieved.

At the same time, it is also important to collect samples with genotype data and detailed clinical information. These samples will be essential to dissect clinical and biological heterogeneity using genetic instruments. Eventually, the achievement of larger sample sizes as compared to those available nowadays may allow to reliably identify genetic loci specifically linked to depression phenotypes defined more strictly. Adoption of a balanced portfolio reconciling the two main strategies discussed in the present review is probably the optimal approach to progress further in unraveling depression genetic architecture.

## Supplementary material

For supplementary material accompanying this paper visit https://doi.org/10.1017/S0033291719002502. click here to view supplementary material
