# Supplementary material for: Unraveling the genetic architecture of major depressive disorder: merits and pitfalls of the approaches used in genome-wide association studies
Source: Psychol Med. 2019 Sep 27;49(16):2646–56. doi: 10.1017/S0033291719002502 (PMC6877467; doi:10.1017/S0033291719002502)
Supplement: Supplementary file 1 [file S0033291719002502sup001.docx]

**Supplementary material**

This document contains supplementary material for the article *Unraveling the Genetic Architecture of Major Depression Disorder: Merits and Pitfalls of the Approaches Used in Genome-Wide Association Studies* by Schwabe, I., Milaenschi, P.F., Gerring, Z., Sullivan, P.F., Schulte, E., Suppli, N.P., Thorp, J.G., Derks, E.M. and Middeldorp, C.M.

**Supplementary Table 1**. Overview of all Genome Wide Association studies on depression

| **Study** | **Population** | **Depression phenotype/s** | **Ascertainment** | | | **Total N** | **N cases** | **N controls** | **GWS loci** | **H^2^_SNP_ (s.e.)** |
| --- | --- | --- | --- | --- | --- | --- | --- | --- | --- | --- |
|  |  |  | **Clinical diagnosis / diagnostic interview** | **Self-reported diagnosis / treatment** | **Self-reported questionnaires / symptoms** |  |  |  |  |  |
| Sullivan et al. (2009) | European | MDD | x |  |  | 3,540 | 1,738 | 1,802 | 0 | ***** |
| Lewis et al. (2010) | European | Recurrent MDD | x |  |  | 3,230 | 1,636 | 1,594 | 0 | * |
| Muglia et al. (2010) | European | Recurrent MDD | x |  |  | 3,141 | 1,359 | 1,782 | 0 | * |
| Rietschel et al. (2010) | European | MDD | x |  |  | 1,968 | 604 | 1,364 | 0 | * |
| Shi et al. (2011) | European | Recurrent, early-onset MDD | x |  |  | 2,656 | 1,020 | 1,636 | 0 | * |
| Shyn et al. (2011) | European | MDD (all cases)  Recurrent, early-onset MDD | x  x |  |  | 7,385  5,619 | 3,957  2,191 | 3,428  3,428 | 0  0 | *  * |
| Kohli et al. (2011) | European | MDD | x |  | x | 15,089 | 4,088 | 11,001 | 0 | * |
| Wray et al. (2012) | European | MDD | x |  |  | 12,664 | 5,763 | 6,901 | 0 | * |
| Ripke et al. (2013) | European | MDD | x |  |  | 18,759 | 9,240 | 9,519 | 0 | 0.21 (0.021) |
| Hek et al. (2013) | European | Depressive symptoms |  |  | x | 34,549 | - | - | 0 | * |
| Cai et al. (2015) | Han Chinese women | Recurrent MDD | x |  |  | 10,640 | 5,303 | 5,337 | 2 | 0.21 (0.030) |
| Okbay et al. (2016) | European | Depressive symptoms | x |  | x | 180,866 | 16,471 | 58,835 | 2 | 0.04 (0.004) |
| Hyde et al. (2016) | European | Major depression | x | x |  | 478,240 | 130,620 | 347,620 | 15 | 0.06 (*) |
| Direk et al. (2017) | European | Broad depression  (meta-analysis of MDD and depressive symptoms) | x |  | x | 70,017 | 9,240 | 9,519 | 1 | 0.30 (0.040) |
| Power et al. (2017) | European | Age at onset stratified MDD:  Late-onset (adult-onset) MDD | x  x |  |  | 18,439  ~13,519  (octiles 5-8) | 8,920  ~4,000 | 9,519  9,519 | 1 | 0.23 (0.046) |
| Mbarek et al. (2017) | European | MDD | x |  |  | 6,507 | 1,941 | 4,565 | 1 | * |
| Milaneschi et al. (2017) | European | MDD (all cases)  MDD with increased appetite/weight  MDD with decreased appetite/weight  MDD with no change in appetite/weight | x  x  x  x |  |  | 26,628  16,662  20,138  18,212 | 11,837  1,871  5,347  3,421 | 14,791  14,791  14,791  14,791 | na  0  0  na | 0.14 (0.08)  0.11 (0.03)  0.11 (0.02)  0.08 (0.02) |
| Hall et al. (2018) | European | Major depression (all cases)  Recurrent major depression  Male major depression  Female major depression | x  x  x  x | x  x  x  x | x  x  x  x | 43,062  39,556  19,886  23,169 | 10,851  7,345  3,852  6,997 | 32,211  32,211  16,034  16,172 | 0  0  1  0 | 0.12 (0.02)  0.12 (0.02)  0.13 (0.03)  0.05 (0.03) |
| Howard et al. (2018) | European | Help-seeking for mental health difficulties (Broad depression)  Probable MDD  MDD (ICD-coded) | x | x  x | x | 322,580  174,519  217,584 | 113,769  30,603  8,276 | 208,811  143,916  209,308 | 14  2  1 | 0.10 (0.004)  0.05 (0.006)  0.10 (0.012) |
| Wray et al. (2018) | European | Major depression | x | x | x | 461,134 | 135,458 | 344,901 | 44 | 0.09 (0.004) |
| Peterson et al. (2018) | Han Chinese women | MDD (all cases)  MDD with exposure to adversity  MDD with no exposure to adversity  *(adversity assessed through stressful life events and childhood abuse questionnaires)* | x  x  x |  |  | 9,599  2,628  6,971 | 4,785  1,646  3,139 | 4,814  982  3,832 | 0  0  3 | 0.31 (0.037)  0.34 (0.159)  0.38 (0.048) |
| Xiaoyan et al. (2018) | European (n = 326,113) and Han Chinese women (n = 10,640) | Major depression | x | x |  | 336,753 | 90,150 | 246,603 | 10 | * |
| Dunn et al. (2018) | Hispanic / Latino | Depressive symptoms  Depressive symptoms adjusted for anti-depressant use  Depressive symptoms excluding anti-depressant users |  |  | x  x  x | 12,310  12,310  11,486 | -  -  - | -  -  - | 0  0  0 | 0.04 (0.031)  0.03 (0.031)  0.04 (0.033) |
| Howard et al. (2019) | European | Major depression | x | x | x | 807,553 | 246,363 | 561,190 | 101 | 0.09 (0.003) |
| Cai et al. (2018) | European | Help-seeking from psychiatrist  Help-seeking from GP  Probable MDD  Self-reported MDD  DSM-based major depression  Recurrent DSM-based major depression |  | x  x  x  x | x  x  x | 333,412  332,622  79,575  253,919  67,171  59,385 | 36,286  113,260  21,117  19,805  16,301  10,302 | 297,126  219,362  58,398  234,114  50,870  49,083 | 5  24  0  0  1  0 | 0.13 (0.018)  0.14 (0.008)  0.18 (0.015)  0.11 (0.009)  0.26 (0.022)  0.32 (0.026) |

*not reported

**Additional references for supplementary Table**

Cai, N., Bigdeli, T. B., Kretzschmar, W., Li, Y., Liang, J., Song, L., . . . Hu, Z. (2015). Sparse whole-genome sequencing identifies two loci for major depressive disorder. *Nature, 523*(7562), 588.

Cai, N., Kendler, K., & Flint, J. (2018). Minimal phenotyping yields GWAS hits of low specificity for major depression. *bioRxiv*. doi: 10.1101/440735

Cathryn M. Lewis, Mandy Y. Ng, Amy W. Butler, Sarah Cohen-Woods, Rudolf Uher, Katrina Pirlo, . . . Peter McGuffin. (2010). Genome-Wide Association Study of Major Recurrent Depression in the U.K. Population. *American Journal of Psychiatry, 167*(8), 949-957. doi: 10.1176/appi.ajp.2010.09091380

Direk, N., Williams, S., Smith, J. A., Ripke, S., Air, T., Amare, A. T., . . . Sullivan, P. F. (2017). An Analysis of Two Genome-wide Association Meta-analyses Identifies a New Locus for Broad Depression Phenotype. *Biological Psychiatry, 82*(5), 322-329. doi: https://doi.org/10.1016/j.biopsych.2016.11.013

Dunn, E. C., Sofer, T., Wang, M. J., Soare, T. W., Gallo, L. C., Gogarten, S. M., . . . Smoller, J. W. (2018). Genome-wide association study of depressive symptoms in the Hispanic Community Health Study/Study of Latinos. *J Psychiatr Res, 99*, 167-176. doi: 10.1016/j.jpsychires.2017.12.010

Hall, L. S., Adams, M. J., Arnau-Soler, A., Clarke, T.-K., Howard, D. M., Zeng, Y., . . . McIntosh, A. M. (2018). Genome-wide meta-analyses of stratified depression in Generation Scotland and UK Biobank. *Translational Psychiatry, 8*, 9. doi: 10.1038/s41398-017-0034-1

Hek, K., Demirkan, A., Lahti, J., Terracciano, A., Teumer, A., Cornelis, M. C., . . . Murabito, J. (2013). A genome-wide association study of depressive symptoms. *Biol Psychiatry, 73*(7), 667-678. doi: 10.1016/j.biopsych.2012.09.033

Howard, D. M., Adams, M. J., Clarke, T.-K., Hafferty, J. D., Gibson, J., Shirali, M., . . . McIntosh, A. M. (2019). Genome-wide meta-analysis of depression in 807,553 individuals identifies 102 independent variants with replication in a further 1,507,153 individuals. *Nature Neuroscience*

Howard, D. M., Adams, M. J., Shirali, M., Clarke, T.-K., Marioni, R. E., Davies, G., . . . McIntosh, A. M. (2018). Genome-wide association study of depression phenotypes in UK Biobank identifies variants in excitatory synaptic pathways. *Nature Communications, 9*(1), 1470. doi: 10.1038/s41467-018-03819-3

Hyde, C. L., Nagle, M. W., Tian, C., Chen, X., Paciga, S. A., Wendland, J. R., . . . Winslow, A. R. (2016). Identification of 15 genetic loci associated with risk of major depression in individuals of European descent. *Nature Genetics, 48*, 1031. doi: 10.1038/ng.3623

Kohli, Martin A., Lucae, S., Saemann, Philipp G., Schmidt, Mathias V., Demirkan, A., Hek, K., . . . Binder, Elisabeth B. (2011). The Neuronal Transporter Gene SLC6A15 Confers Risk to Major Depression. *Neuron, 70*(2), 252-265. doi: https://doi.org/10.1016/j.neuron.2011.04.005

Li, X., Luo, Z., Gu, C., Hall, L. S., McIntosh, A. M., Zeng, Y., . . . the 23andMe Research, T. (2018). Common variants on 6q16.2, 12q24.31 and 16p13.3 are associated with major depressive disorder. *Neuropsychopharmacology, 43*(10), 2146-2153. doi: 10.1038/s41386-018-0078-9

Mbarek, H., Milaneschi, Y., Hottenga, J.-J., Ligthart, L., de Geus, E. J., Ehli, E. A., . . . Boomsma, D. I. (2017). Genome-wide significance for PCLO as a gene for major depressive disorder. *Twin Research and Human Genetics, 20*(4), 267-270.

Milaneschi, Y., Lamers, F., Peyrot, W. J., Baune, B. T., Breen, G., Dehghan, A., . . . the Major Depressive Disorder Working Group of the Psychiatric Genomics, C. (2017). Genetic Association of Major Depression With Atypical Features and Obesity-Related Immunometabolic Dysregulations. *JAMA Psychiatry, 74*(12), 1214-1225. doi: 10.1001/jamapsychiatry.2017.3016

Muglia, P., Tozzi, F., Galwey, N., Francks, C., Upmanyu, R., Kong, X., . . . Rothen, S. (2010). Genome-wide association study of recurrent major depressive disorder in two European case–control cohorts. *Molecular psychiatry, 15*(6), 589.

Okbay, A., Baselmans, B. M. L., De Neve, J.-E., Turley, P., Nivard, M. G., Fontana, M. A., . . . Cesarini, D. (2016). Genetic variants associated with subjective well-being, depressive symptoms, and neuroticism identified through genome-wide analyses. *Nature Genetics, 48*, 624. doi: 10.1038/ng.3552

Peterson, R. E., Cai, N., Dahl, A. W., Bigdeli, T. B., Edwards, A. C., Webb, B. T., . . . Kendler, K. S. (2018). Molecular Genetic Analysis Subdivided by Adversity Exposure Suggests Etiologic Heterogeneity in Major Depression. *Am J Psychiatry, 175*(6), 545-554. doi: 10.1176/appi.ajp.2017.17060621

Power, R. A., Tansey, K. E., Buttenschøn, H. N., Cohen-Woods, S., Bigdeli, T., Hall, L. S., . . . Lewis, C. M. (2017). Genome-wide Association for Major Depression Through Age at Onset Stratification: Major Depressive Disorder Working Group of the Psychiatric Genomics Consortium(). *Biological Psychiatry, 81*(4), 325-335. doi: 10.1016/j.biopsych.2016.05.010

Rietschel, M., Mattheisen, M., Frank, J., Treutlein, J., Degenhardt, F., Breuer, R., . . . Cichon, S. (2010). Genome-Wide Association-, Replication-, and Neuroimaging Study Implicates HOMER1 in the Etiology of Major Depression. *Biological Psychiatry, 68*(6), 578-585. doi: https://doi.org/10.1016/j.biopsych.2010.05.038

Ripke, S., Wray, N. R., Lewis, C. M., Hamilton, S. P., Weissman, M. M., Breen, G., . . . Sullivan, P. F. (2013). A mega-analysis of genome-wide association studies for major depressive disorder. *Mol Psychiatry, 18*(4), 497-511. doi: 10.1038/mp.2012.21

Shi, J., Potash, J. B., Knowles, J. A., Weissman, M. M., Coryell, W., Scheftner, W. A., . . . Levinson, D. F. (2011). Genome-wide association study of recurrent early-onset major depressive disorder. *Molecular psychiatry, 16*, 193. doi: 10.1038/mp.2009.124

Shyn, S. I., Shi, J., Kraft, J. B., Potash, J. B., Knowles, J. A., Weissman, M. M., . . . Hamilton, S. P. (2011). Novel loci for major depression identified by genome-wide association study of Sequenced Treatment Alternatives to Relieve Depression and meta-analysis of three studies. *Molecular psychiatry, 16*, 202. doi: 10.1038/mp.2009.125

Sullivan, P. F., de Geus, E. J. C., Willemsen, G., James, M. R., Smit, J. H., Zandbelt, T., . . . Penninx, B. W. J. H. (2009). Genome-wide association for major depressive disorder: a possible role for the presynaptic protein piccolo. *Molecular psychiatry, 14*, 359. doi: 10.1038/mp.2008.125

Wray, N. R., Pergadia, M. L., Blackwood, D. H., Penninx, B. W., Gordon, S. D., Nyholt, D. R., . . . Sullivan, P. F. (2012). Genome-wide association study of major depressive disorder: new results, meta-analysis, and lessons learned. *Mol Psychiatry, 17*(1), 36-48. doi: 10.1038/mp.2010.109

Wray, N. R., Ripke, S., Mattheisen, M., Trzaskowski, M., Byrne, E. M., Abdellaoui, A., . . . Sullivan, P. F. (2018). Genome-wide association analyses identify 44 risk variants and refine the genetic architecture of major depression. *Nature Genetics*. doi: 10.1038/s41588-018-0090-3
